# Identification of key binding site residues of MCT1 for AR-C155858 reveals the molecular basis of its isoform selectivity

**DOI:** 10.1042/BJ20141223

**Published:** 2015-02-06

**Authors:** Bethany Nancolas, Richard B. Sessions, Andrew P. Halestrap

**Affiliations:** *School of Biochemistry, University of Bristol, Medical Sciences Building, University Walk, Bristol BS8 1TD, U.K.

**Keywords:** lactate transport, molecular dynamics (MD) simulations, molecular modelling, monocarboxylate transporter 1 (MCT1), monocarboxylate transporter 4 (MCT4), *Xenopus* oocytes, CHC, α-cyano-4-hydroxycinnamate, DIDS, 4,4′-diisothiocyano-2,2′-stilbenedisulfonic acid, MCT, monocarboxylate transporter, MD, molecular dynamics, OPLS–AA, optimized potentials for liquid simulations–all atoms, POPC, 1-palmitoyl-2-oleoyl-sn-glycero-3-phosphocholine, SDM, site-directed mutagenesis, SPC, simple point charge, TM, transmembrane helix, WT, wild-type

## Abstract

The proton-linked monocarboxylate transporters (MCTs) are required for lactic acid transport into and out of all mammalian cells. Thus, they play an essential role in tumour cells that are usually highly glycolytic and are promising targets for anti-cancer drugs. AR-C155858 is a potent MCT1 inhibitor (*K*_i_ ~2 nM) that also inhibits MCT2 when associated with basigin but not MCT4. Previous work [Ovens, M.J. et al. (2010) Biochem. J. 425, 523–530] revealed that AR-C155858 binding to MCT1 occurs from the intracellular side and involves transmembrane helices (TMs) 7–10. In the present paper, we generate a molecular model of MCT4 based on our previous models of MCT1 and identify residues in the intracellular substrate-binding cavity that differ significantly between MCT4 and MCT1/MCT2 and so might account for differences in inhibitor binding. We tested their involvement using site-directed mutagenesis (SDM) of MCT1 to change residues individually or in combination with their MCT4 equivalent and determined inhibitor sensitivity following expression in *Xenopus* oocytes. Phe^360^ and Ser^364^ were identified as important for AR-C155858 binding with the F^360^Y/S^364^G mutant exhibiting >100-fold reduction in inhibitor sensitivity. To refine the binding site further, we used molecular dynamics (MD) simulations and additional SDM. This approach implicated six more residues whose involvement was confirmed by both transport studies and [^3^H]-AR-C155858 binding to oocyte membranes. Taken together, our data imply that Asn^147^, Arg^306^ and Ser^364^ are important for directing AR-C155858 to its final binding site which involves interaction of the inhibitor with Lys^38^, Asp^302^ and Phe^360^ (residues that also play key roles in the translocation cycle) and also Leu^274^ and Ser^278^.

## Abstract 2

A combination of molecular modelling, site-directed mutagenesis and molecular dynamics simulations define the binding site of MCT1 for AR-C155858, a potent and specific inhibitor. Key amino acids within the binding site differ between MCT1 and MCT4 accounting for isoform specificity.

## INTRODUCTION

Almost all mammalian cells require L-lactic acid to be transported across their plasma membranes. Some tissues, such as white muscle, rely on glycolysis for most of their ATP production even under normoxic conditions and thus produce large quantities of lactic acid that must be transported out of the cells. Other tissues take up lactic acid for oxidation as a respiratory fuel (e.g. heart, red muscle fibres and neurons) or for gluconeogenesis (liver and kidney) and lipogenesis (adipose tissue) [1,2]. In all cases, lactic acid must be rapidly transported across the plasma membrane and this is mediated by proton-linked monocarboxylate transporters (MCTs) that in mammals are the part of a family of related transporter proteins containing 14 members. Of these, only MCTs 1–4 have been confirmed as lactic acid transporters and their properties and tissue distribution have been extensively characterized [3,4]. MCT1 is present in the majority of cells and most strongly expressed in those that oxidize lactic acid such as heart and red skeletal muscle fibres. By contrast, MCT4 is strongly expressed only in tissues that rely on glycolysis, such as white muscle, but is up-regulated in all cells in response to hypoxia coincident with the up-regulation of glycolysis [2,4]. Indeed, characterization of the kinetics of MCT4 reveals that it is especially suited for mediating export of lactic acid produced by glycolysis because, unlike MCT1, it has a very low affinity for pyruvate. This ensures that only lactate and not pyruvate is lost from the cell, which is important to ensure the NADH generated by glycolysis can be reoxidized [5,6]. The distribution of MCT2 is less well conserved between species, but it is most often expressed in tissues that require high affinity lactic acid uptake for gluconeogenesis (liver and kidney) or oxidation (neurons). This is consistent with its higher affinity for L-lactate than MCT1 [2,4,7]. MCT3 is less well characterized but its expression is limited to the retinal pigment epithelium and choroid plexus where it acts to mediate lactic acid efflux [4,8].

Rapidly dividing cells such as tumour cells and proliferating T-lymphocytes are highly glycolytic even under aerobic conditions, a phenomenon first recognized by Otto Warburg and subsequently referred to as ‘the Warburg effect’ [9–12]. It is now known that tumour cells express large amounts of either or both MCT1 and MCT4 [13], with the most aggressive tumours often expressing mainly MCT4 which is up-regulated by over-expression of hypoxia-inducible factor 1α (HIF-1α) [14–17]. Furthermore, within solid tumours there may be heterogeneity in lactate metabolism, with the hypoxic centre producing lactic acid that is exported via MCT4 to be taken up for oxidation by the better oxygenated peripheral cells that express more MCT1 [18,19]. Soon after the discovery that lactic acid efflux from cells is carrier-mediated, it was recognized that inhibition of this process was a potential target for chemotherapy [20,21] but the lack of a specific inhibitor prevented realization of this goal. α-cyano-4-hydroxycinnamate (CHC), the inhibitor first used to confirm that lactate transport was carrier-mediated, has been employed for this purpose [22]. However, this is not a good candidate because it also inhibits mitochondrial pyruvate transport and, hence, glucose oxidation with a potency of at least two orders of magnitude greater than MCT1 [4,23]. Nevertheless, CHC was shown to kill hypoxic tumour cells [18] and this sparked a renewed interest in MCTs as a cancer target. A major breakthrough in this approach came from the development by AstraZeneca of a novel class of MCT1-specific inhibitors [24]. These agents exhibit *K*_i_ values in the low nanomolar range and are inactive against MCT4 at concentrations up to 10 μM [25,26]. They were originally developed as immunosuppressive drugs that potently inhibit proliferation of the highly glycolytic T-lymphocytes by binding to MCT1 and preventing lactic acid efflux [24,26]. Subsequently, their role as potential anti-cancer drugs was explored and AR-C155858 was confirmed to inhibit growth of Ras-transformed fibroblasts [27] whereas AZD-3965 is now in clinical trials targeting tumours that show predominant expression of MCT1 [28]. However, many highly aggressive tumours predominantly express MCT4 and are not sensitive to inhibition of MCT1 [15–17]. Rather, MCT4-specific inhibitors are required and development of these would be greatly assisted if the binding site of MCT1 for AR-C155858 could be identified. Such information might allow the structural differences between MCT1 and MCT4 to be correlated with key residues involved in inhibitor binding which could then be modified to target MCT4 specifically.

MCTs are members of the larger solute carrier family and are predicted to have 12 transmembrane helices (TMs) with the C- and N-termini facing the cytosol and a large intracellular loop between TMs 6 and 7 [29]. They are not glycosylated themselves, but they do require association with a member of the basigin family which comprises three related single membrane spanning glycoproteins that contain two or three extracellular immunoglobulin folds [29,30]. Basigin itself is the usual partner for MCT1, MCT3 and MCT4 [31,32] whereas embigin is the more usual partner for MCT2 [33], although both basigin and neuroplastin can fulfil this role [34]. Although no 3D X-ray crystal structures are available for MCTs, we have used molecular modelling in combination with site-directed mutagenesis (SDM) to develop a probable structure of MCT1 in an inward open conformation [35] and a second structure in an outward facing conformation containing the bound inhibitor, 4,4′-diisothiocyano-2,2′-stilbenedisulfonic acid (DIDS) [36]. We subsequently employed chimeras of MCT1 and MCT4 to demonstrate that the binding site of MCT1 for AR-C155858 involves TMs 7–10 of the C-terminal domain of MCT1 [25]. We also showed that inhibition of MCT2 by AR-C155858 depends on the ancillary protein to which MCT2 is bound, with inhibition observed only when it is associated with basigin (which is unusual) but not when it is associated with embigin. These effects of the ancillary protein are mediated by interactions of the C-terminus of embigin with the C-terminus and TMs 3 and 6 of MCT2 [37].

In the present paper, we generate and compare new molecular models of MCT1 and MCT4 that allow us to identify key residues in the cavity of MCT1 in the inward facing conformation that differ between MCT1 and MCT4 and so might account for differences in inhibitor binding. We then test their involvement with SDM and further refine the binding site using a combination of molecular dynamics (MD) simulations and additional SDM. This approach also provides information on the probable pathway taken by the inhibitor to access its final binding site.

## EXPERIMENTAL

### Materials

*Xenopus laevis* oocytes were purchased from the Xenopus Resource Centre. All reagents were obtained from Sigma unless otherwise stated. Polyclonal antibodies against the C-terminal 16 amino acids of rat MCT1 and MCT4 were raised in rabbits as described previously [38,39] whereas anti-rabbit secondary antibodies for immunofluorescence microscopy were from Jackson ImmunoResearch. [^14^C]-L-lactate (0.5 mM, 3.7 MBq/ml) was obtained from PerkinElmer. AR-C155858 and [^3^H]-AR-C155858 were obtained from AstraZeneca.

### Methods

#### Site-directed mutagenesis

SDM of MCT1 and MCT4 within the oocyte expression vector (pGHJ) was performed using a QuickChange kit (Stratagene) as described previously [35]. Primers containing the desired mutation were designed between 20 and 40 bases in length with a melting point >78°C and GC content >40% (sequences provided in Supplementary Table S1). For PCR, they were added at a final concentration of 125 ng/μl together with plasmid DNA at a final concentration of 0.2 ng/μl in a final reaction volume of 50 μl. Thermocycling was performed for 16 cycles: 30 s at 95°C, 60 s at 55°C, 4.5 min at 68°C. The presence of the correct mutation was confirmed by sequencing (The Sequencing Service, University of Dundee).

#### Measurement of MCT transport activity in *X. laevis* oocytes

Measurement of MCT transport activity in *X. laevis* oocytes was performed essentially as described previously [35,37]. cRNA was prepared by *in vitro* transcription using the mMessage mMachine kit (Ambion) from the appropriate linearized pGHJ plasmid. cRNA (10 ng) was injected into oocytes in a final volume of 13.8 nl. Controls received the equivalent volume of water. Oocytes were cultured for three days in oocyte recipe 3 (OR3) medium composed of Leibovitz L-15 medium (Life technologies, 200 ml), sterile water (136.6 ml), 100× penicillin/streptomycin (3.4 ml), fungizone (0.25 μg/ml) and tetracycline (100 μg/ml). Rates of L-lactate transport in the absence and presence of increasing concentrations of AR-C155858 were determined by measuring [^14^C]-L-lactate uptake over 5 min, the time over which uptake was linear with time [25,37]. Confirmation of plasma membrane expression was provided by Western blotting of membrane fractions and immunofluorescence microscopy of oocyte sections, as described previously [25,40]

#### Measurement of radiolabelled inhibitor binding in *X. laevis* oocytes

Ten MCT-expressing oocytes were washed briefly in assay buffer [75 mM NaCl, 2 mM KCl, 0.82 mM MgCl_2_, 1 mM CaCl_2_ and 20 mM MES (pH 6.0)] before incubation in 3 ml of assay buffer containing a total concentration of 50 nM AR-C155858 ([^3^H]-AR-C155858 at a specific activity of 933 Bq/pmol) for 45 min at room temperature. Control oocytes were treated similarly to calculate non-specific membrane binding of AR-C155858. Oocytes were then washed once in ice-cold assay buffer (5 ml) and membranes were isolated in the presence of protease inhibitors, as described previously [40]. The membrane pellet was solubilized in SDS (10% in water, 100 μl) by vigorous vortexing and [^3^H] content assayed by scintillation counting. The amount of [^3^H] remaining in the supernatant was always low (<2% of the membrane fraction) indicating that most of the inhibitor was bound under these conditions.

#### Molecular modelling

The homology model of MCT4 was created using a sequence alignment (Clustal Omega) [41] to MCT1 and building the model using the MCT1 homology model previously created [35] as a template. The large intracellular loop between helices 6 and 7 was removed during development of the model, as it is not thought to contribute to either inhibitor binding or substrate transport [25]. Insight II (Accelrys Inc.) was used for visualization and Discover 2.98 (Accelrys) was used to minimize the energy of the resulting structures. The intracellular loop and C-terminal tail were added to the MCT1 homology models before simulation. The loop was created with a random conformation using the loop-building function of Insight II as these regions are not predicted to adopt a particular structure due to the high variation in their sequence and length between isoforms. In addition, we have shown previously that the C-terminal tail of MCT1 is not involved in AR-C155858 inhibition [25]. Coordinates for all the model structures generated in the present study are available as PDB files in Supplementary data.

#### Molecular docking studies

GoldSuite (http://www.ccdc.cam.ac.uk, [42]) was used to dock the AstraZeneca inhibitor, AR-C155858 into the channel of the MCT models. The program was given a defined atom as a starting position and a distance within which poses were sampled [typically 10 Å (1 Å=0.1 nm)]. The 100 best solutions were saved; other parameters were kept as default. The GoldScore scoring method was used to rank the best solutions.

#### Molecular dynamics simulations

GROMACS (version 4.6.5) was used for MD simulations. The OPLS–AA (optimized potentials for liquid simulations–all atoms) force field [43] was used along with the SPC (simple point charge) water model [44] and Berger lipids [45]. The ligand AR-C155858 was parameterized with GAFF (general amber force field) [46] using antechamber [47] and acpype [48] to generate GROMACS/OPLS–AA compatible data. The following general protocol was applied to each homology model and protein–ligand complex. The model was aligned in a membrane of 512 POPC (1-palmitoyl-2-oleoyl-sn-glycero-3-phosphocholine) lipids (downloaded from http://www.wcm.ucalgary.ca/tieleman/downloads/popc128a.npdb) in a box 12×12×12 nm and solvated with SPC water. The protein was embedded using the GROMACS utility g_membed. Ions (Na^+^ and Cl^−^) were added to the solvent using the genion utility at a physiological concentration of 0.15 mM and to attain overall system neutrality. Energy minimization was performed using the steepest decent algorithm for 2000 steps followed by position-restraint dynamics (5 ns in length) restraining the protein to the initial coordinates. After the position-restraint run, an unconstrained dynamics simulation was performed for 50–200 ns at a temperature of 300 K as an isothermal–isobaric (NPT) ensemble under periodic boundary conditions using the PME (particle mesh Ewald) method for long-range electrostatics. Coordinates were saved every 100 ps and energies every 2 ps with a step-size of 2 fs. MD simulations were performed using the high-performance computing facility at the University of Bristol Advanced Computing Research Centre. GROMACS analysis tools were used to analyse the simulations. Simulations were typically viewed in VMD (version 1.8.6) [49] and graph data exported to Microsoft Excel for plotting.

## RESULTS

### Docking of AR-C155858 to the MCT1 homology model in an inward-open conformation identifies two residues important for inhibition

**Figure 1:**
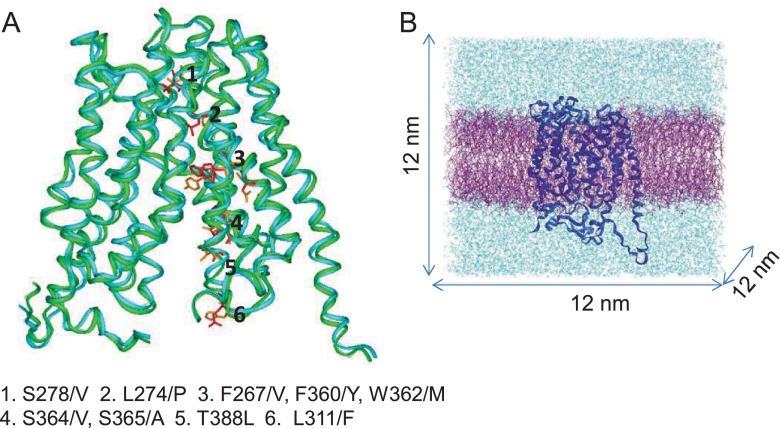
Residue differences between MCT1 and MCT4 isoforms within TM helices 7–10 (**A**) Cα atoms of MCT4 homology model (green) are super-imposed on to the model of MCT1 (blue). Residues lying in predicted TM helices 7–10 which differ between isoforms are coloured in red (MCT1) and their equivalent in MCT4 in orange and their positions indicated. (**B**) Example of a simulation set-up. The model of MCT1 (dark blue) is embedded in a POPC bilayer (purple) and solvated to a box size of 12 nm^3^ in water containing 0.15 M NaCl (light blue).

Previous work using chimeras of MCT1 and MCT4 indicated that the binding site for AR-C155858 lies in TMs 7–10 of the C-terminal domain of MCT1 and is accessed from the inside of the cell [25]. This would suggest that the conformation to which AR-C155858 binds is likely to be one with a channel open to the cytosol and usually termed ‘inward-open’. Two previous models of MCT1 have been generated; one in the inward-open conformation based on the crystal structure of the glycerol-3-phosphate transporter (GlpT) [35] and another in an intermediate conformation that is open to both the intracellular and extracellular milieu. The latter is suggested to be the conformation to which the inhibitor DIDS binds [36]. We first generated a model of MCT4 in the inward-open conformation (MCT4_IO). This was created by alignment of the MCT1 and MCT4 sequences followed by building the MCT1 model on the previously established MCT1 model (MCT1_IO), in turn based on the crystal structure of GlpT. We then looked for residues in the cavity that were in the vicinity of the proposed substrate-binding site and differed between MCT1 and MCT4 as shown in Figure 1A. Several residues were identified and, of these, Phe^360^ in MCT1 (Tyr^336^ in MCT4) stood out because mutation of this to cysteine in MCT1 allows the transporter to accommodate larger substrates such as mevalonate [36,50]. In addition, we have shown that mutation of Phe^360^ to alanine prevents transport of lactate by MCT1 (Supplementary Figure S1). Consequently, in both the proposed conformations of MCT1 described above, AR-C155858 was docked into the channel using GOLD with Phe^360^–CZ given as the starting atom and the inhibitor was allowed to roam a distance of 10 Å (1 Å=0.1 nm) in each direction. The best scoring pose was then taken as the starting structure for a MD simulation by embedding the complex in a solvated POPC lipid bilayer (Figure 1B), as described in Methods.

**Figure 2:**
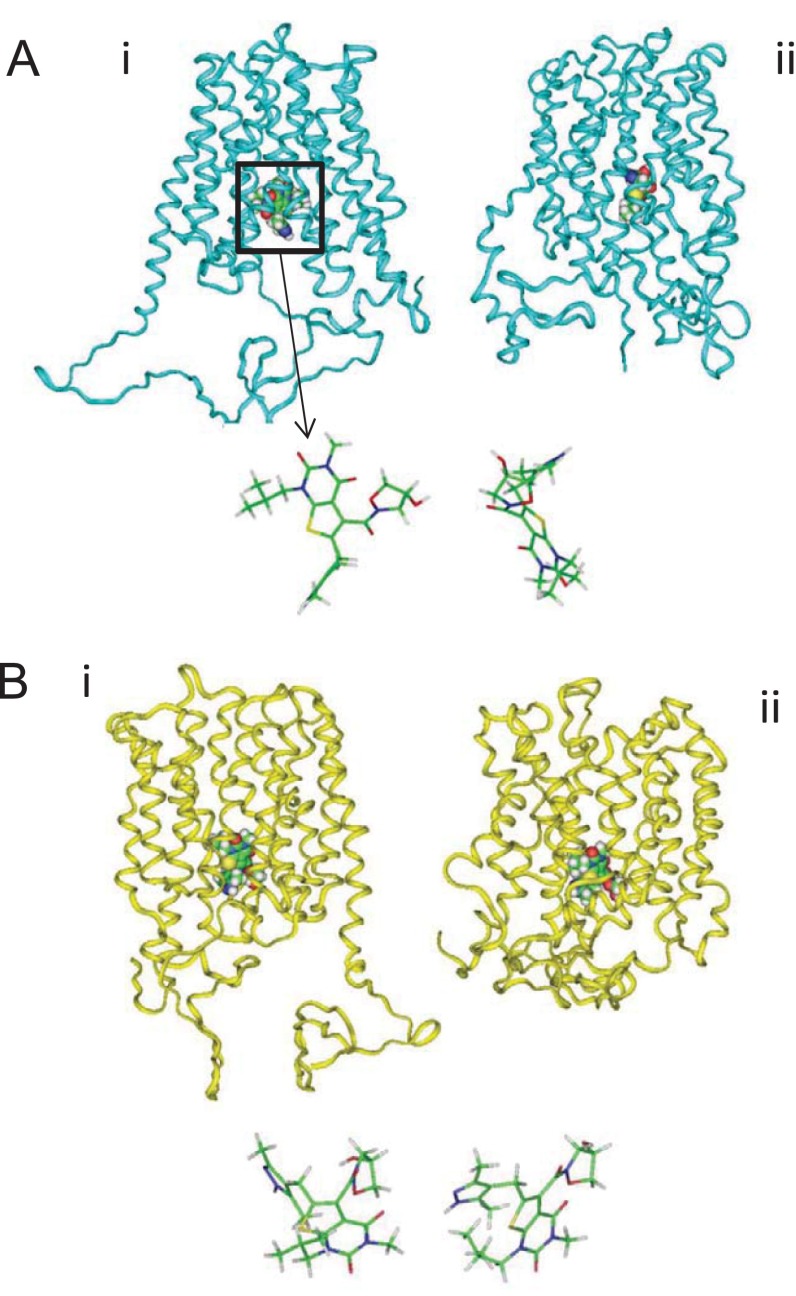
Docking of AR-C155858 to MCT1 Docking to the inward (**A**) and intermediate (**B**) conformations of MCT1 before (i) and after (ii) simulation using GOLD. The positions of AR-C155858 are shown in stick form to demonstrate the change in pose over 160–200 ns.

**Figure 3:**
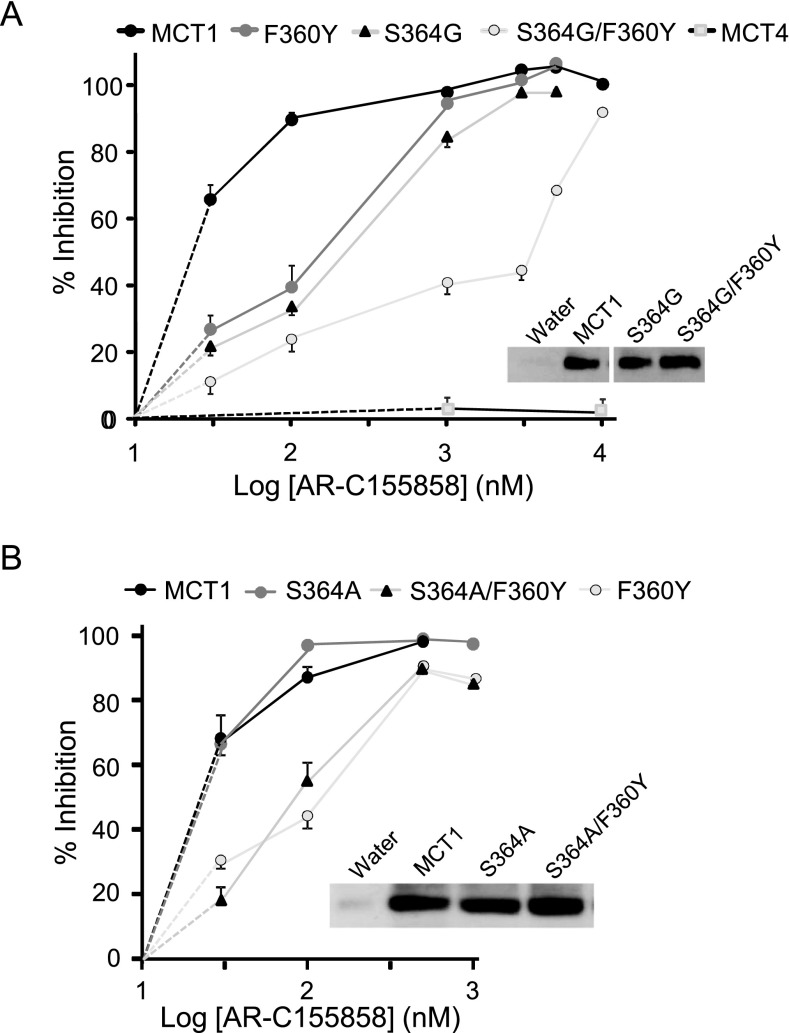
Inhibition of the S^364^G/F^360^Y and S^364^A/F^360^Y mutants Inhibition of L-lactate transport by mutants expressed in *Xenopus* oocytes. Uptake was corrected for MCT-independent transport in water-injected oocytes (*n*=5) and data are presented as percentage inhibition ±% S.E.M. (error bars) for S^364^G/F^360^Y (**A**) and S^364^A/F^360^Y (**B**). For each inhibitor concentration the number of oocytes used was 15–64 (**A**) or 15–25 (**B**) with fewer oocytes used at the higher inhibitor concentrations (>500 nM) where variation between oocytes was less. Data for MCT4 are included in (**A**) for comparison. Note that where no error bars are shown they lie within the data symbol. Plasma membrane expression of the MCTs was determined by Western blotting of membrane fractions using the C-terminal MCT1 antibody.

When the MD simulation was started using the inward-open conformation of MCT1, AR-C155858 migrated from its initial docked position to a different position within the MCT1 channel (Figure 2A), but did not form any hydrogen bonds with surrounding residues within the cavity during the simulation (Table 1). Rather, hydrophobic contacts appeared to mediate the interactions between protein and inhibitor during the simulation, inconsistent with specific inhibitor binding. However, when the simulations were repeated using the intermediate conformation of MCT1 (MCT1_Int) as the starting point, little movement from the initial position was observed, indicating a strong interaction of AR-C155858 with MCT1 at this location (Figure 2B). Here, multiple residues lay in close proximity forming hydrogen bonds with AR-C155858 (Table 1). The amino acids surrounding the inhibitor in this docked position were mutated to their equivalent in MCT4 (as determined by sequence alignment) or, if the same in MCT4, to alanine and the modified MCT1 constructs expressed in oocytes for measurement of AR-C155858 inhibition of L-lactate transport. Mutation of three residues (S^364^G and F^360^Y in TM 10 and N^147^A in TM 5) were found to decrease significantly the inhibition by AR-C155858 at 30 nM (Supplementary Figure S2A) whereas inhibition by AR-C155858 at 100 nM was only decreased in the S^364^G and F^360^Y mutants. Using a wider range of inhibitor concentrations it was confirmed that individual mutation of F^360^Y and S^364^G significantly decreased inhibition by AR-C155858 at concentrations up to 100 nM, and when both residues were mutated (S^364^G/F^360^Y) inhibition remained diminished at concentrations up to 5 μM (Figure 3A). Further investigation showed that for Ser^364^ it was the deletion of the entire side chain to glycine rather than the removal of the hydroxy group that mediated the reduced inhibition. Thus the S^364^A mutant displayed similar inhibition to wild-type (WT) MCT1 whereas the double S^364^A/F^360^Y mutant behaved similarly to the single F^360^Y mutant (Figure 3B). We confirmed that all the mutants were expressed at similar levels in the plasma membrane (as shown by the illustrative Western blots) and displayed similar lactate uptake over a 5 min time period (Supplementary Table S2). Taken together, these data suggest that Phe^360^ and Ser^364^ play important roles in AR-C155858 binding to MCT1 and their absence in MCT4 provides some explanation as to why MCT4 is insensitive to the inhibitor. However, even when both these residues are mutated to their counterparts in MCT4, significant inhibition is observed at 10 μM which is not the case for MCT4. This implies that additional residues play a role in AR-C155858 binding, some of which may provide further specificity of the inhibitor for MCT1 over MCT4.

**Table 1:** Residues forming hydrogen bonds with AR-C155858 during simulation in the inward-open or intermediate conformation of MCT1 Residues were identified using the GROMACS utility g_hbond over the final 40 ns of the simulations (allowing for equilibration).

Simulation starting point	Simulation time (ns)	Residues forming hydrogen bonds with AR-C155858 in last 40 ns
Inward-open	200	None
Intermediate	164	Tyr^34^, Asn^129^, Asn^147^, Arg^306^, Ser^364^, Leu^367^, Glu^391^

We then sought to confirm the involvement of these residues in AR-C155858 inhibition of MCT1 by mutation of the equivalent residues in MCT4 to match those present in MCT1. The expectation was that this would enhance inhibition of MCT4 by AR-C155858. However, only one of these mutants, Y^336^F, transported lactate at a substantial rate (Supplementary Figure S3A). Nevertheless, this mutant showed a significant increase in sensitivity to inhibition at 10 μM AR-C155858 compared with WT MCT4, supporting the involvement of Phe^360^ in MCT1 inhibition (Supplementary Figure S3B).

### Measurement of [^3^H]-AR-C155858 binding indicates essential residues in MCT1 transport also have a role in inhibition

**Figure 4:**
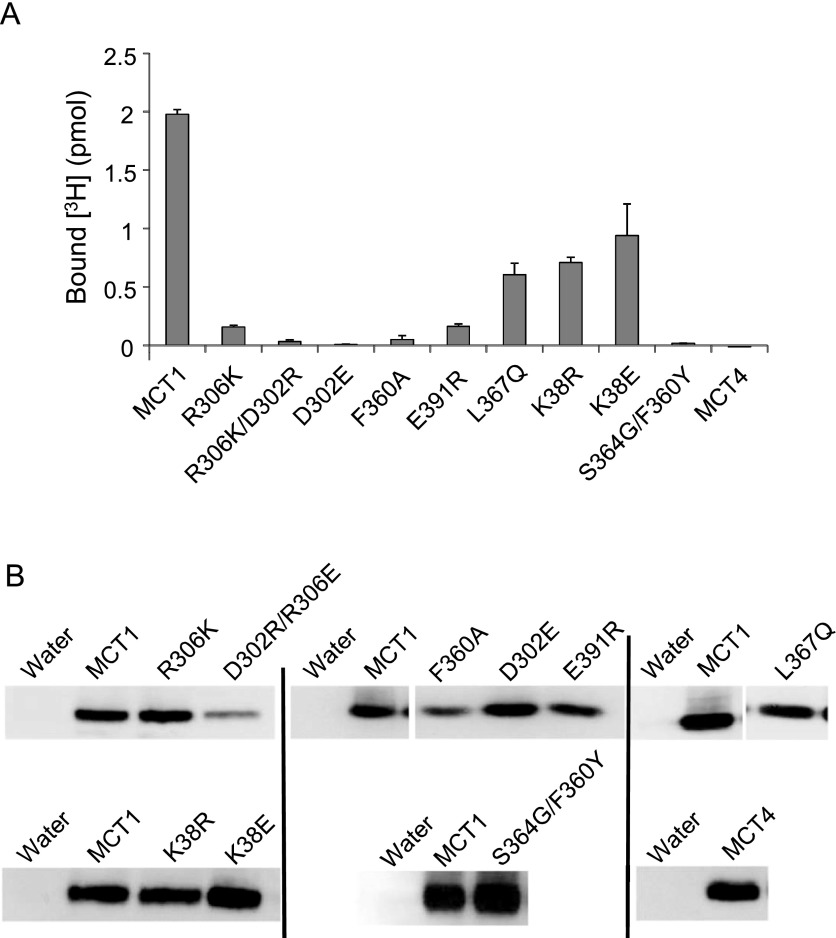
Binding of [^3^H]-AR-C155858 to MCT1 and MCT1-mutants (**A**) Oocytes were injected with RNA or water 3 days prior to incubation of 20 oocytes in 3 ml of pH 6.0 transport buffer containing 50 nM AR-C155858 (specific activity 933 Bq/pmol [^3^H]-AR-C155858) for 45 min at room temperature. Membranes were then isolated and the pellet containing the membrane fraction solubilized in 20% SDS before scintillation counting. Non-specific binding was corrected for by subtracting the bound inhibitor for water-injected oocytes and data are shown as means±S.E.M. of 20–50 individual oocytes from 2–5 separate experiments. (**B**) Plasma membrane proteins (10 μg) were separated by SDS/PAGE and MCT1 expression detected by Western blot using the C-terminal MCT1 antibody. Equal loading was confirmed by Coomassie staining of the PVDF membrane. Black lines indicate separate blots.

During the MD simulation of inhibitor binding in the intermediate conformation described above, hydrogen bonds were formed between the oxygen atoms of the isoxazolidine group of the inhibitor and the guanidinium group of Arg^306^. We have previously shown that this residue is essential for transport and that it is likely to form a charge pair with Asp^302^, another essential residue [35]. We have proposed that these two residues, together with Lys^38^, play a key role in the translocation of lactate and its accompanying proton [36]. For this reason, it was not possible to explore the role of Arg^306^ in AR-C155858 binding by measuring inhibition of transport because even conservative mutation of this residue to lysine rendered the transporter inactive, despite it being well expressed at the plasma membrane [36]. To overcome this problem, we utilized a radiolabelled form of AR-C155858 to measure inhibitor binding to plasma membrane preparations from *Xenopus* oocytes expressing the different MCT1 mutants. Data are shown in Figure 4. We first confirmed that binding of 70 nM [^3^H]-AR-C155858 was totally blocked in the S^364^G/F^360^Y mutant as predicted from the transport studies. We also demonstrated that conservative mutation of Arg^306^ to lysine or a charge swap between Asp^302^ and Arg^306^ (D^302^R/R^306^E) resulted in much lower binding despite similar plasma membrane expression of the MCT1, as did mutation of Asp^302^ alone. Other mutations in MCT1 that still showed normal membrane expression but no transport activity were also assessed for their ability to bind AR-C155858 as shown in Figure 4. Of these, K^38^E/R, F^360^A, L^367^Q and E^391^R also displayed reduced inhibitor binding to some degree and thus are likely to play some role in AR-C155858 binding. However, this may not reflect a direct interaction with the inhibitor in the final bound state but rather prevent the conformational changes required to generate this state, effectively ‘locking’ MCT1 in a conformation inaccessible to AR-C155858.

### Neither inward-open nor intermediate structures of MCT1 can explain involvement of all residues implicated in binding

**Figure 5:**
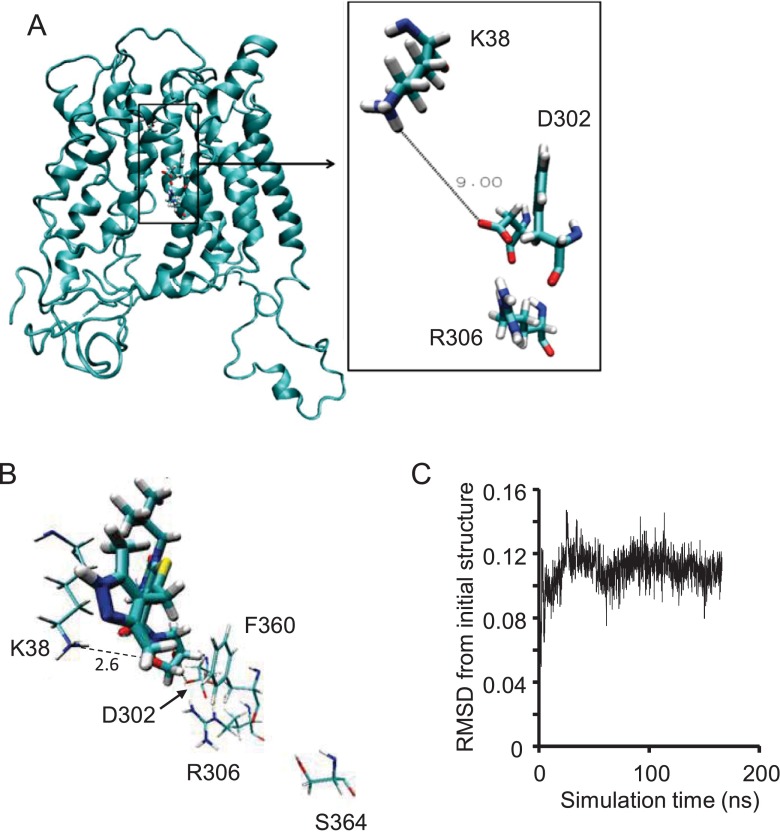
Inward-intermediate conformation of MCT1 taken from a structure observed during the lactate simulation (**A**) Conformation of MCT1 after dynamic simulation for 200 ns with lactate present in the substrate channel. Lactate interacted with Arg^306^ and Lys^38^ during this simulation but has been removed from the resulting structure for simulation with inhibitor. Inset shows the distance between Lys^38^ and Asp^302^ at the end of the simulation. (**B**) Position of AR-C155858 after 200 ns simulation when docked initially to a 10 Å radius around Lys^38^. Lys^38^, Asp^302^, Phe^360^, Arg^306^ and Ser^364^ are shown. (**C**) RMSD of all AR-C155858 atoms versus or with respect to inward-intermediate simulation time.

**Figure 6:**
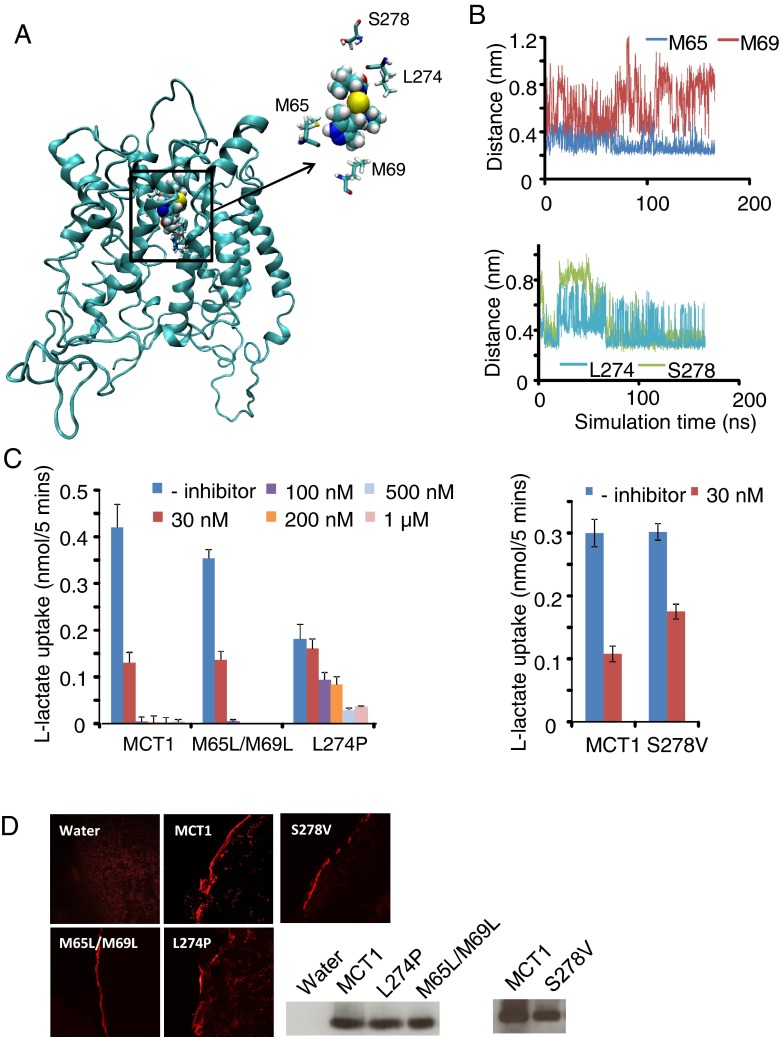
The role of residues in close proximity to AR-C155858 during the inward-intermediate simulation in inhibitor binding (**A**) The position of residues Met^65^, Met^69^, Leu^274^ and Ser^278^ relative to AR-C155858 is shown after 200 ns simulation in the inward-intermediate conformation of MCT1. (**B**) The distance between AR-C155858 and residues Met^65^, Met^69^, Leu^274^ and Ser^278^ during the time course of the simulation. Distances shown are between atoms: M^65^S to AR-C–HG^1^; M^69^CE to AR-C–His^121^; L^274^HG to AR-C–SE^1^ and S^278^OG to AR-C–His^103^. (**C**) Inhibition of lactate uptake by M^65^L/M^69^L, L^274^P and S^278^V mutants in response to increasing concentrations of AR-C155858. (**D**) Plasma membrane expression of the MCT1 mutants shown by immunofluorescence microscopy and Western blot using the C-terminal MCT1 antibody.

**Figure 7:**
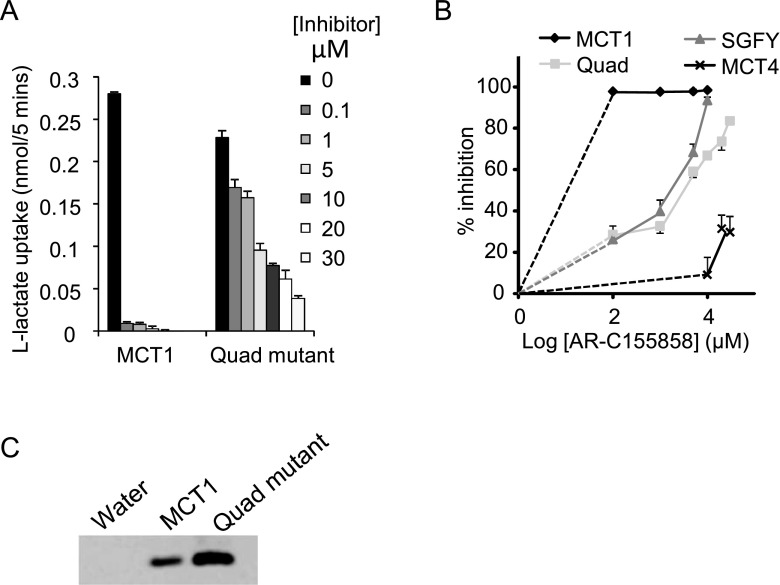
Inhibition of a quadruple mutant of MCT1 by AR-C155858 (**A**) Absolute rates of L-lactate uptake by oocytes expressing WT and the quadruple mutant of MCT1 (L^274^P/S^278^V/F^360^Y/S^364^G) at increasing concentrations of AR-C155858. Data are presented as mean±S.E.M (error bars) of 5–37 separate oocytes. Fewer oocytes were used for higher AR-C155858 concentrations due to the smaller variation in lactate uptake observed. All data are corrected for uptake by water-injected oocytes (*n*=5). Note that where no error bars are shown, they lie within the data symbol. (**B**) The same data are expressed for the percentage inhibition of transport and additional data are shown for the double mutant S^364^G/F^360^Y and for WT MCT4. (**C**) Plasma membrane expression of the MCTs is shown by Western blotting using the C-terminal MCT1 antibody.

Since docking and simulation of AR-C155858 binding to either of the two individual conformations of MCT1 alone cannot explain the involvement of Phe^360^, Ser^364^, Arg^306^, Asp^302^ and Lys^38^ in mediating inhibition, it is likely that some residues are critically important in structural changes that direct AR-C155858 to its final binding site. During MD simulations of MCT1 starting from the inward open conformation, the structure becomes more compact with a kink in helix 1. This is probably enabled by both the conserved motif in this helix, which is abundant in glycine residues, and the conserved proline (Pro^37^) that precedes Lys^38^. This leads to movement of Lys^38^ into the channel of MCT1 and decreases the distance between Asp^302^ and Lys^38^ to only 9 Å to give the structure shown in Figure 5A (MCT1_II). Docking of AR-C155858 into a site defined by a 10 Å radius around Lys^38^ (atom NZ) indicated hydrogen bonding with both Lys^38^ and Asp^302^ simultaneously and close proximity to the ring of Phe^360^ (Figure 5B). Subsequent MD simulation of this structure showed a stable conformation over 200 ns, consistent with a strong binding site (Figure 5C). It should be noted that Lys^38^ and Asp^302^ are conserved in MCT4 which does not bind AR-C155858 tightly and thus other residues must be important in determining inhibitor specificity. Four other residues implicated in hydrogen bonding during the inward-intermediate simulation (Leu^274^, Ser^278^, Met^65^ and Met^69^) do differ between the isoforms (Figures 6A and 6B). The mutation of Leu^274^ to proline (its equivalent in MCT4) resulted in significantly (*P*<0.05) reduced inhibition at concentrations up to 200 nM (Figure 6C) and this was confirmed by the determination of radiolabelled inhibitor binding which was reduced to 10% of that observed for WT MCT1 (result not shown). The mutation of Ser^278^ to valine (present in MCT4) also reduced inhibition at 30 nM AR-C155858 by about 50% without any effect on activity or expression (Figures 6C and 6D). Little, if any, effect of mutation of both Met^65^ and Met^69^ to leucine (the equivalent residues in MCT4) was observed. We finally created a mutant containing all four mutations identified as important for AR-C155858 binding. As expected, addition of mutations L^274^P and S^278^V to the S^364^G/F^360^Y mutant further decreased inhibition at higher AR-C155858 concentrations (Figures 7A and 7B). The activity of this mutant was also lower than WT MCT1, as demonstrated by the slightly decreased control lactate uptake compared with MCT1, with its overexpression at the plasma membrane when compared with MCT1 (Figure 7C). Taken together, these results suggest that in addition to Phe^360^, Ser^364^, Lys^38^, Asp^302^ and Arg^306^, there is an involvement of Leu^274^ and, possibly, Ser^278^ in mediating inhibitor binding, but probably not Met^65^ and Met^69^.

### Simulations with the *R*-enantiomer of AR-C155858 also support the predicted binding position

**Figure 8:**
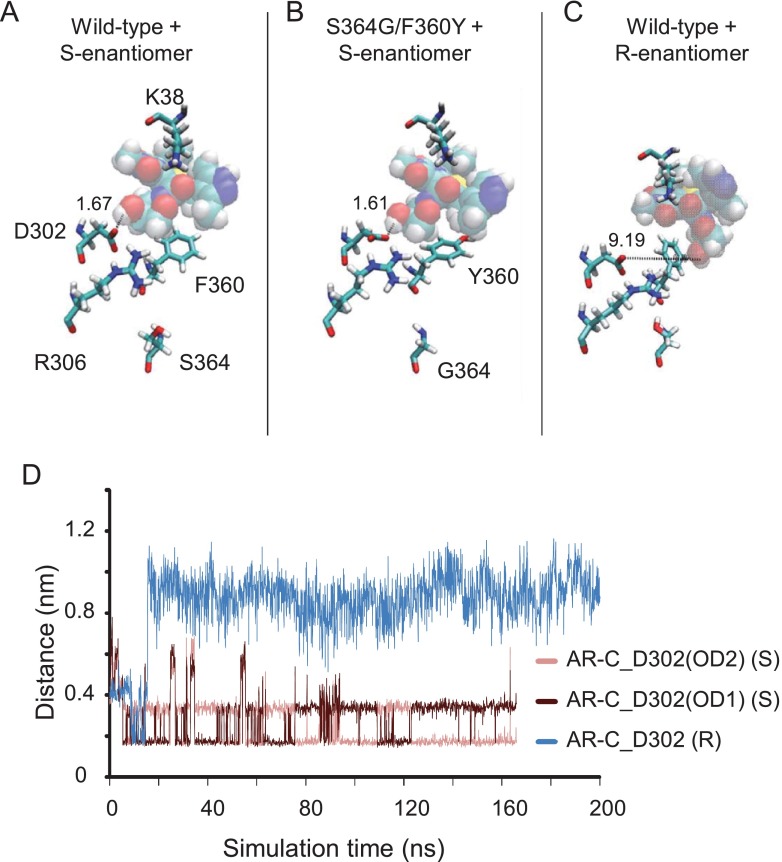
Position of AR-C155858 after 167–200 ns simulation in the WT MCT1 or S^364^G/F^360^Y mutant *S*-AR-C155858 was docked into the inward-intermediate conformation of MCT1 and the best scoring solution taken for simulation (167–200 ns). The same initial position and conformation of AR-C155858 was used as the start for each simulation shown. (**A**) The position of *S*-AR-C155858 after 167 ns simulation in the WT MCT1 model. The hydrogen bond between Asp^302^ and AR-C155858 hydroxy group is indicated (1.67 Å). (**B**) The position of *S*-AR-C155858 after simulation in a model containing the mutations S^364^G and F^360^Y (indicated). (**C**) The position of *R*-AR-C155858 after simulation in the WT MCT1 model. (**D**) The distance between AR-C hydroxy group and D^302^ oxygen atoms (OD1 and OD2) in the simulation with the *S*-enantiomer (pink) and *R*-enantiomer (blue).

**Figure 9:**
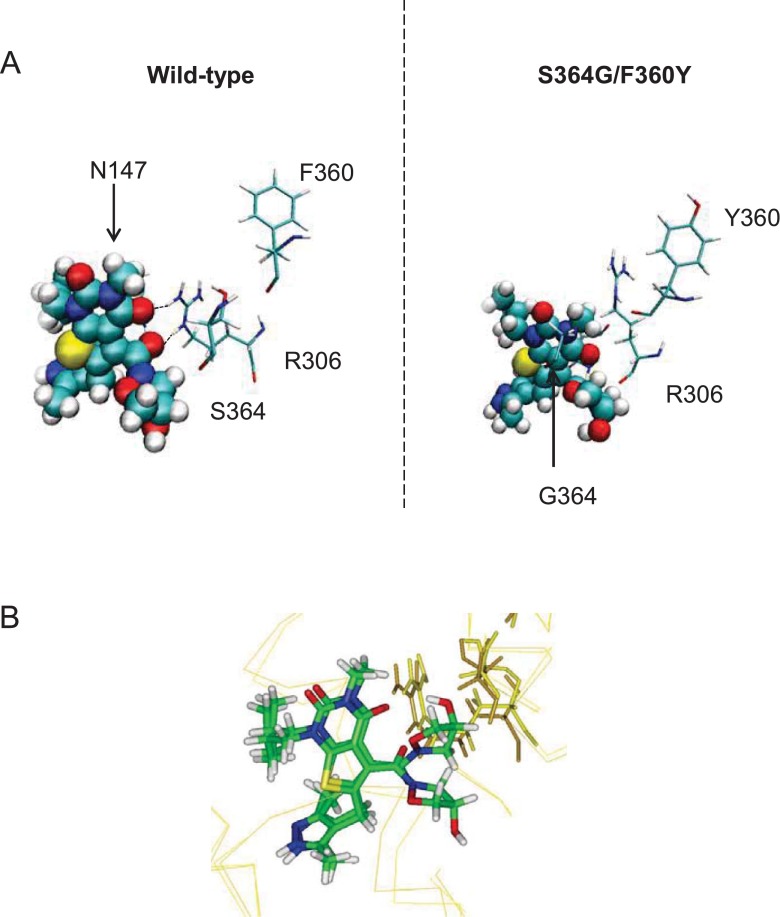
Position of AR-C155858 after simulation in the intermediate conformation (**A**) *S*-AR-C155858 was docked into the intermediate conformation of MCT1 and the best scoring solution taken for simulation in the WT model (left, 200 ns) or MCT1 model containing the mutations S^364^G and F^360^Y (right, 164 ns). (**B**) The position of *S*-AR-C155858 or *R*-AR-C155858 after 200 ns simulation in the WT MCT1 model. The same initial position and conformation of AR-C155858 was used as the start for all simulations.

Further support for the proposed binding site was provided by MD simulations with the *R*-enantiomer of AR-C155858 which is more than two orders of magnitude less potent at inhibiting MCT1 in the Jurkat cell line (AstraZeneca, unpublished data). The *R*- or *S*-enantiomer was placed in the inward-intermediate conformation of MCT1 in the position indicated in Figure 5 (MCT1_II) and simulations of 160 ns and 200 ns respectively were run to assess the stability of this binding position. The position of the *S*-enantiomer was found to be stable during the simulation, with the hydroxy group forming a persistent hydrogen bond with Asp^302^ throughout (Figures 8A and 8D). By contrast, although a hydrogen bond formed briefly between the *R*-enantiomer and Asp^302^, this was lost after only 14 ns as the drug moved to a position further towards the N-domain of MCT1 (Figures 8B and 8D). Interestingly, if the simulation was repeated with the S^364^G/F^360^Y mutant of MCT1 (that shows greatly reduced inhibitor binding) no differences were observed compared with the WT (Figure 8C). This again suggests that there are residues involved in directing the inhibitor to its final binding site that are distinct from those involved in the final high-affinity binding site. In the present model, the difference in the affinity of MCT1 for the *R*- and *S*-enantiomers of AR-C155858 is accounted for by the latter process rather than the former. In support of this hypothesis, simulation of the *S*-enantiomer in the fully-intermediate conformation (MCT1_Int) results in a final position lacking the hydrogen bonds formed during simulation with WT MCT1 model (Figure 9A), indicating that a site including Arg^306^ and Asn^147^ may represent a transitional binding site, also involved in directing the inhibitor toward its final binding site. Here too, simulation with the *R*-enantiomer does not result in a different binding position (Figure 9B).

## DISCUSSION

### Proposed binding site for AR-C155858

Our data implicate Asp^302^, Arg^306^, Lys^38^, Leu^274^, Ser^278^, Phe^360^ and Ser^364^ in the tight binding of AR-C155858 to MCT1 and the binding site shown in Figure 6 can explain the involvement of all of these residues. Ser^364^ does not appear to interact directly with the inhibitor in its final binding site even though its mutation to glycine reduces inhibition. However, this mutation may act by allowing more flexibility to helix 10, so preventing the correct positioning along the helix of Phe^360^ or other residues that do interact directly with the inhibitor. The failure of MD simulations to reproduce the lower affinity of the mutant MCT1 for AR-C155858 does not undermine this conclusion since it is possible that the mutation of these residues may alter the structure of the protein sufficiently to prevent access to the predicted binding position. The simulation we used, in which the inhibitor is placed in a predicted binding position at the start, would not be sensitive to this difference. This is especially true for Ser^364^, since this residue is predicted to play a role in helix 10 orientation, allowing correct positioning of Phe^360^. Further support for this hypothesis is gained from a simulation in an intermediate conformation, where simulation of MCT1 containing the double mutation S^364^G/F^360^Y led to a different docking position of AR-C155858 than that found in simulations of WT MCT1. We suggest that this conformation may be required for correct transition of AR-C155858 from a transient binding position involving Asn^147^ and Arg^306^ to a more tightly bound position involving residues Lys^38^, Asp^302^, Leu^274^ and Ser^278^. Further support for these residues playing an important role in inhibitor binding comes from the observation that, when expressed with basigin, MCT2 is inhibited by AR-C155858 with a similar affinity to its inhibition of MCT1 [37]. Table 2 presents a comparison of the residues of the proposed binding site for AR-C155858 in MCT1 with those at the equivalent position in MCT2 and MCT4. It is striking that, unlike for MCT4, all the key binding residues are conserved in MCT2 except for two conservative changes (Leu^274^ to Ile^263^ and Ser^278^ to Ala^273^).

**Table 2:** Amino acid residues associated with the proposed binding site for AR-C155858 Sequences were aligned using ClustalΩ. Residues shown are from rat, but are conserved in each isoform across other species analysed (human, bovine, mouse).

Proposed binding site residue		
MCT1	MCT2	MCT4
Lys^38^	Lys^43^	Lys^40^
Asp^302^	Asp^297^	Asp^278^
Arg^306^	Arg^301^	Arg^282^
Phe^360^	Phe^255^	Tyr^336*^
Ser^364^	Ser^259^	Gly^340*^
Leu^274^	Ile^263^	Pro^250*^
Ser^278^	Ala^273^	Val^254*^
Met^65^	Met^71^	Leu^67*^
Met^69^	Met^75^	Leu^71*^
Asn^147^	Asn^153^	Asn^149^

*Residues show non-conservative changes relative to MCT1.

It is of interest that the binding site of AR-C155858 appears to involve key residues (Arg^306^, Asp^302^ and Lys^38^) previously identified as being critical for lactate transport by MCT1. One explanation for this would be if the inhibitor binds to the external surface of the transporter and then uses the normal translocation cycle to reach its final binding site. However, we have previously shown that AR-C155858 inhibits MCT1 expressed in oocytes when it is micro-injected directly into the cytosol, implying that the inhibitor can reach its binding site from the intracellular side [25]. Furthermore, we showed that when MCT2 is expressed with embigin in oocytes inhibition of lactate transport by AR-C155858 is prevented through a mechanism involving the C-terminus of embigin and the C-terminus of MCT2 [37]. This is also most readily explained if the inhibitor binds to MCT1 and MCT2 exclusively from the intracellular side. Thus the requirement of Arg^306^, Asp^302^ and Lys^38^ for AR-C155858 must lie elsewhere, most probably by providing access for the inhibitor from the cytosol to its optimal binding site.

**Figure 10:**
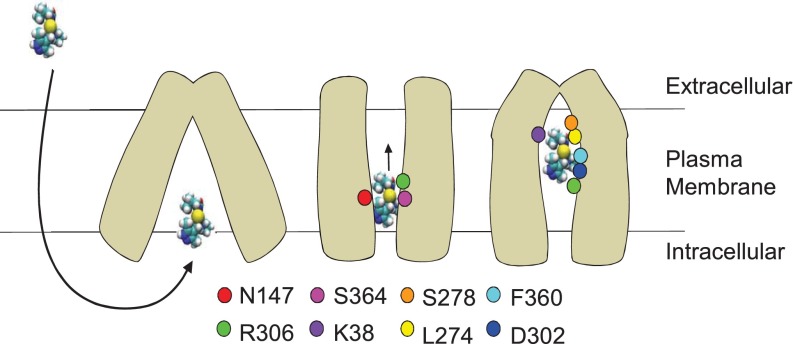
Schematic diagram showing the proposed mechanism of inhibition by AR-C155858 AR-C155858 crosses the plasma membrane to enter MCT1 in the inward-open conformation. An intermediate conformation is adopted, allowing binding of AR-C155858 by interaction with residues in the intracellular half including Asn^147^ (helix 5), Arg^306^ (helix 8) and Ser^364^ (helix 10). A further conformational change then allows movement of AR-C155858 further into the channel of MCT1, interacting with residues in the extracellular half including Lys^38^ (helix 1), Asp^302^ (helix 8), Phe^360^ (helix 10), Lys^274^ and Ser^278^ (helix 7). Note that the *R*- and *S*-enantiomers behave identically in the initial binding (Figure 9B) but only the *S*-enantiomer binds tightly in the final conformation (Figure 8).

It is clear from our studies that a homology model in one conformation cannot fully describe the binding site and that conformational changes are probably involved in providing such access. Hence, we have exploited MD simulations to elucidate the single residue movements that may occur during inhibitor binding and this has provided valuable additional information on the binding mechanism. We suggest that binding starts with a transient binding of AR-C155858 that involves residues toward the cytoplasmic side of MCT1 and that this is required to ‘shuttle’ the inhibitor to the final site indicated in Figure 7. This transient site would utilize residues Asn^147^ and Arg^306^, before the inhibitor moves to a site involving residues Asp^302^, Lys^38^, Leu^274^ and Ser^278^. A schematic diagram indicating this hypothesized double-binding site mode of inhibition is described in Figure 10.

## Online data

Supplementary data
