# Supplementary material for: Identification of key binding site residues of MCT1 for AR-C155858 reveals the molecular basis of its isoform selectivity
Source: Biochem J. 2015 Feb 6;466(Pt 1):177–88. doi: 10.1042/BJ20141223 (PMC4357239; doi:10.1042/BJ20141223)
Supplement: Supplementary data [file bj4660177ntsadd.pdf]

Supplementary Material

Table S1 Primers used in PCR generation of MCT1 mutants.

Mutation	LH primer	RH primer
MCT1: Y34F	catttctattggcttctcctttgcatttcccaaatccatc	gatggatttgggaaatgcaaaggagaagccaatagaaatg
MCT1: T55Y	ttatattcagtgcaacgtacagtgaagtgtcatggatatcgtcc	ggacgatatccatgacacttcactgtacgttgcactgaatataa
MCT1: M65L/M69L	gatatcgtccatcctgctggctgtcctgtatgccggag	ctccggcatacaggacagccagcaggatggacgatatc
MCT1: N129A	cttgctttcaacttggccccagctctgactatg	catagtcagagctggggccaagttgaaagcaag
MCT1: N147A	aagcgaccattggcagcgggactggctatg	catagccagtcccgctgccaatggtcgctt
MCT1: G148A	cgaccattggccaatgccctggctatggcagg	cctgccatagccagggcattggccaatggtcg
MCT1:M151A	gccaatggcctggctgcggcaggcagcccagtg	cactgggctgcctgccgcagccaggccattggc
MCT1: A152L	ccaatggcctggctatgttaggcagcccagtgttcc	ggaacactgggctgcctaacatagccaggccattgg
MCT1: F267V	gtctggaaatgtggtcatggtctttgggctctttacccctttggtc	gaccaaaggggtaaagagcccaaagaccatgaccacatttcc agac
MCT1: F271Y	ggtcatgttctttgggctctatacccctttggtctttc	gaaagaccaaaggggtatagagcccaaagaacatgacc
MCT1: L274P	gggctctttacccctccggtctttcttagtaattatg	cataattactaagaaagaccggaggggtaaagagccc
MCT1: L274P/S278V	gggctctttacccctccggtctttcttgttaattatg	cataattaacaagaaagaccggaggggtaaagagccc
MCT1: S278V	gggctctttacccctttggtctttcttgttaattatggtaagagtaag catttt	aaaatgcttactcttaccataattaacaagaaagaccaaagggg taaagagccc
MCT1: D302E	cattttggctttcgttgaaatggtggcaagacc	ggtcttgccaccatttcaacgaaagccaaaatg
MCT1: F360Y	gtctttggatttgcctatggttggctcagctcc	ggagctgagccaaccataggcaaatccaaagac
MCT1:F360A	gtctttggatttgccgcaggttggctcagctcc	ggagctgagccaacctgcggcaaatccaaagac
MCT1: S364G	gcctttggttggctcggctccgtattgtttgagac	gtctcaaacaatacggagccgagccaaccaaaggc
MCT1: S364G/F360Y	gcctatggttggctcggctccgtattgtttgagac	gtctcaaacaatacggagccgagccaaccataggc
MCT1: S364A	gcctttggttggctcgcctccgtattgtttgagac	gtctcaaacaatacggaggcgagccaaccaaaggc
MCT1: S364A/F360Y	gcctatggttggctcgcctccgtattgtttgagac	gtctcaaacaatacggaggcgagccaaccataggc
MCT1: L367Q	gttggctcagctccgtacagtttgagacgttgatg	catcaacgtctcaaactgtacggagctgagccaac
MCT1: E319R	ggtgaccattgtgagatgttgtcctgtcc	ggacaggacaacatctcacaatggtcacc
MCT1: C392A	ggtgaccattgtggaatgttgtcctgtcctcctg	caggaggacaggacaacattccacaatggtcacc

Table S2 Uninhibited rates of L-lactate uptake into oocytes expressing different MCT1 mutants expressed as a percentage of rates observed for wild-type MCT1 ± S.E.M. of the number of separate oocytes shown. L-lactate uptake was determined over 5 minutes in the absence of AR-C155858 at a lactate concentration of 0.5 mM.

	% of wild type MCT1 activity	n (oocytes)
F360Y	106.9 ± 10.4	25
S364A	111.1 ± 13.1	15
S364A/F360Y	106.7 ± 11.1	16
F360Y	94.8 ± 2.6	153
S364G	98.2 ± 9.1	30
S364G/F360Y	91.3 ± 7.6	83

Figure S1 Expression and transport activity of MCT1 mutant F360A

A: L-lactate uptake was measured over 5 minutes, 3 days after RNA injection. Uptake was corrected for MCT1-independent transport in water-injected oocytes (n = 5) and data are presented as means ± S.E.M. of 10 oocytes for both wild type and mutant MCT1. B: Plasma membrane expression of MCT1 in the oocyte membrane. Crude membrane preparations (10 µg) were separated by SDS-PAGE and MCT1 expressed detected by Western blotting using the MCT1 C-terminal antibody. Equal loading was confirmed by Coomassie staining of the PVDF membrane.

Figure S2 Expression and inhibition of MCT1 mutants

A: AR-C155858 docked to an inward-open conformation of MCT1 identifies residues which may be involved in inhibitor binding. B: Inhibition of MCT1 mutants at 30 nM and 100 nM AR-C155858. Results are taken from 1-7 separate experiments for each mutant. Data are shown as a mean percentage inhibition normalised to the percent of wild-type MCT1 inhibition obtained during the same experiment. Results are shown ± S.E.M. of 20-100 oocytes. Data has been corrected for uptake in water-injected oocytes (n = 5). B: Plasma membrane expression of MCT1 mutants. Crude membrane preparations (10 µg) were separated by SDS-PAGE and MCT1 expressed detected by Western blotting using the MCT1 C-terminal antibody. Equal loading was confirmed by Coomassie staining of the PVDF membrane. Black lines separate different blots. Controls (water injected oocytes) were always negative and have not been shown for each individual blot.

Figure S3 Expression and inhibition of MCT4 mutants

A: L-lactate uptake into *Xenopus* oocytes was measured over 5 minutes, 3 days after RNA injection. Uptake was corrected for MCT4-independent transport in water-injected oocytes (n=5) and data are presented as Means ± S.E.M. of 10-20 oocytes for both wild type and mutant MCT4. Plasma membrane expression of MCT4 in the oocyte membrane is shown in the inset. B: Inhibition of Y336F mutant by 10 µM AR-C155858. Results are shown as % lactate uptake of oocytes incubated for 45 minutes with 10 µM AR-C155858 compared to oocytes incubated in buffer alone. Data are presented as Means ± % S.E.M. of 50-80 oocytes.

FIGURE S1

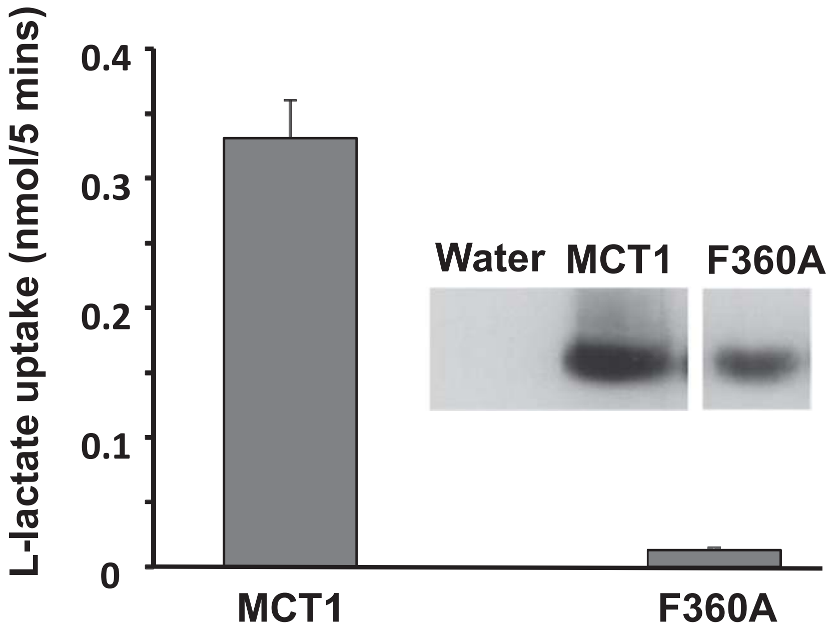
L-lactate uptake (nmol/5 mins)
0.4
0.3
0.2
0.1
0
Water
MCT1
F360A
MCT1
F360A

FIGURE S2

A

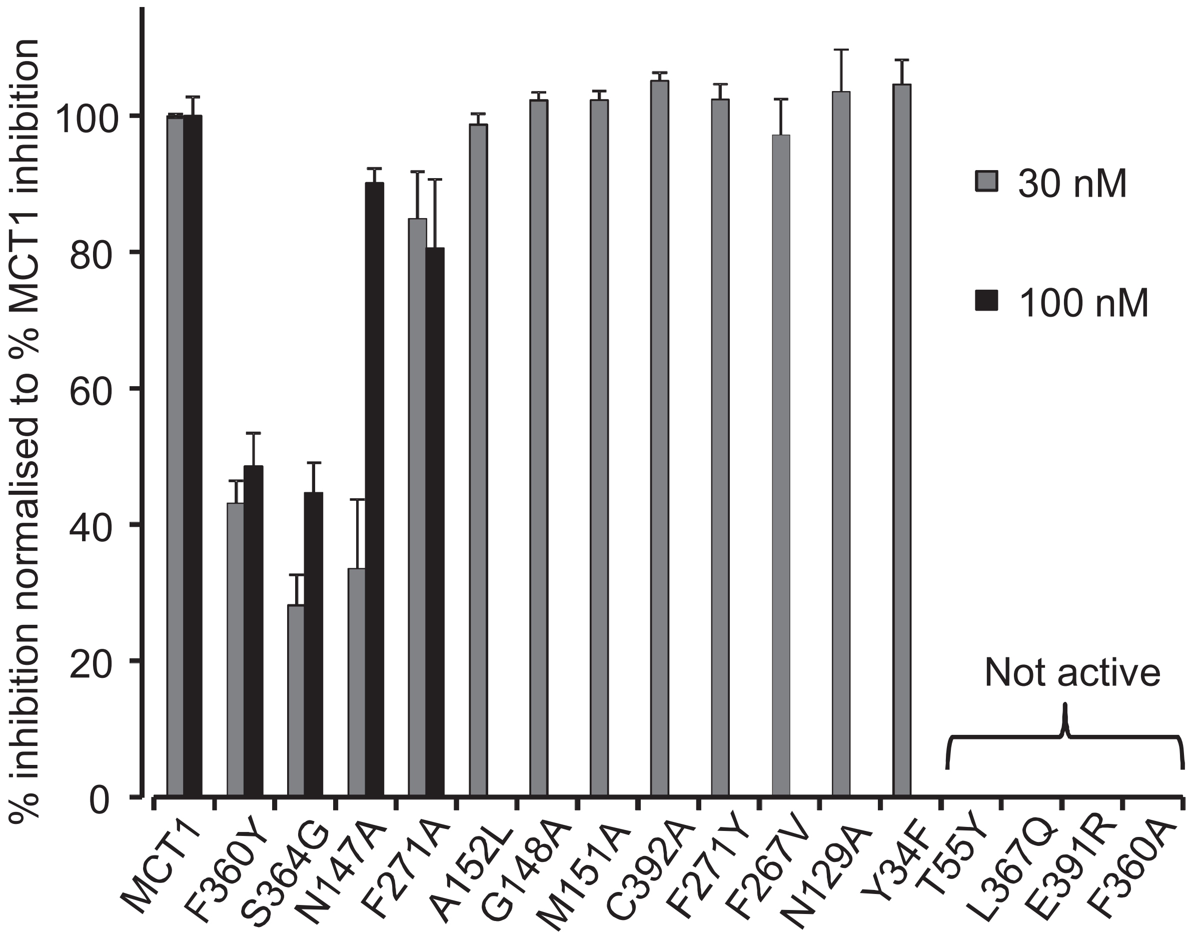
% inhibition normalised to % MCT1 inhibition
100
80
60
40
20
0
30 nM
100 nM
Not active
MCT1
F360Y
S364G
N147A
F271A
A152L
G148A
M151A
C392A
F271Y
F267V
N129A
Y34F
T55Y
L367Q
E391R
F360A

B

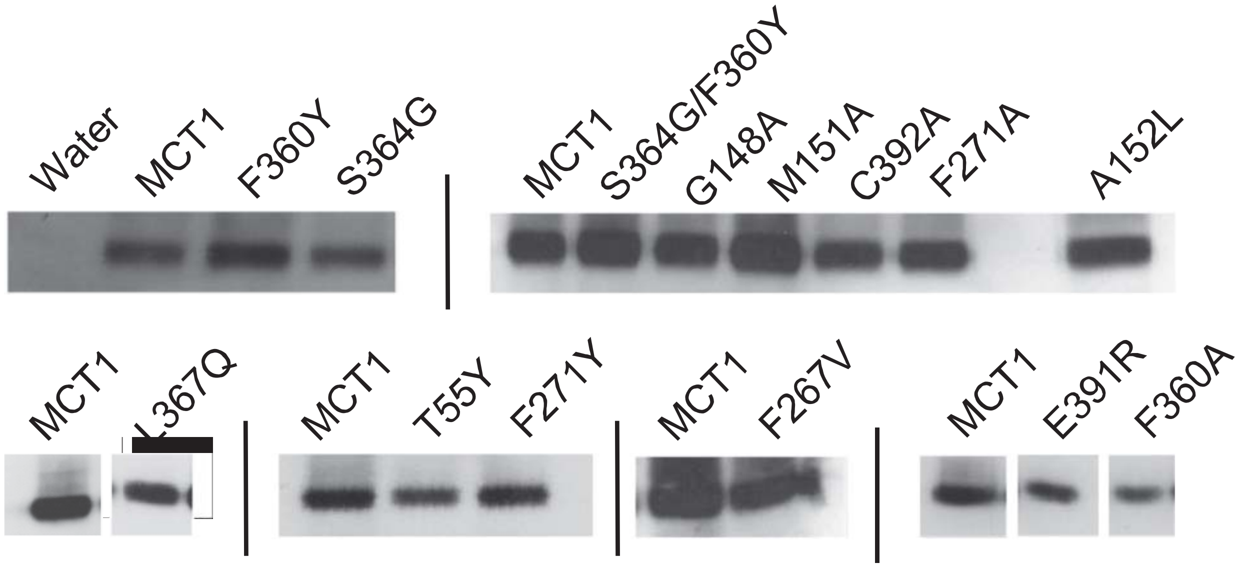
Water
MCT1
F360Y
S364G
MCT1
S364G/F360Y
G148A
M151A
C392A
F271A
A152L
MCT1
L367Q
MCT1
T55Y
F271Y
MCT1
F267V
MCT1
E391R
F360A

FIGURE S3

A

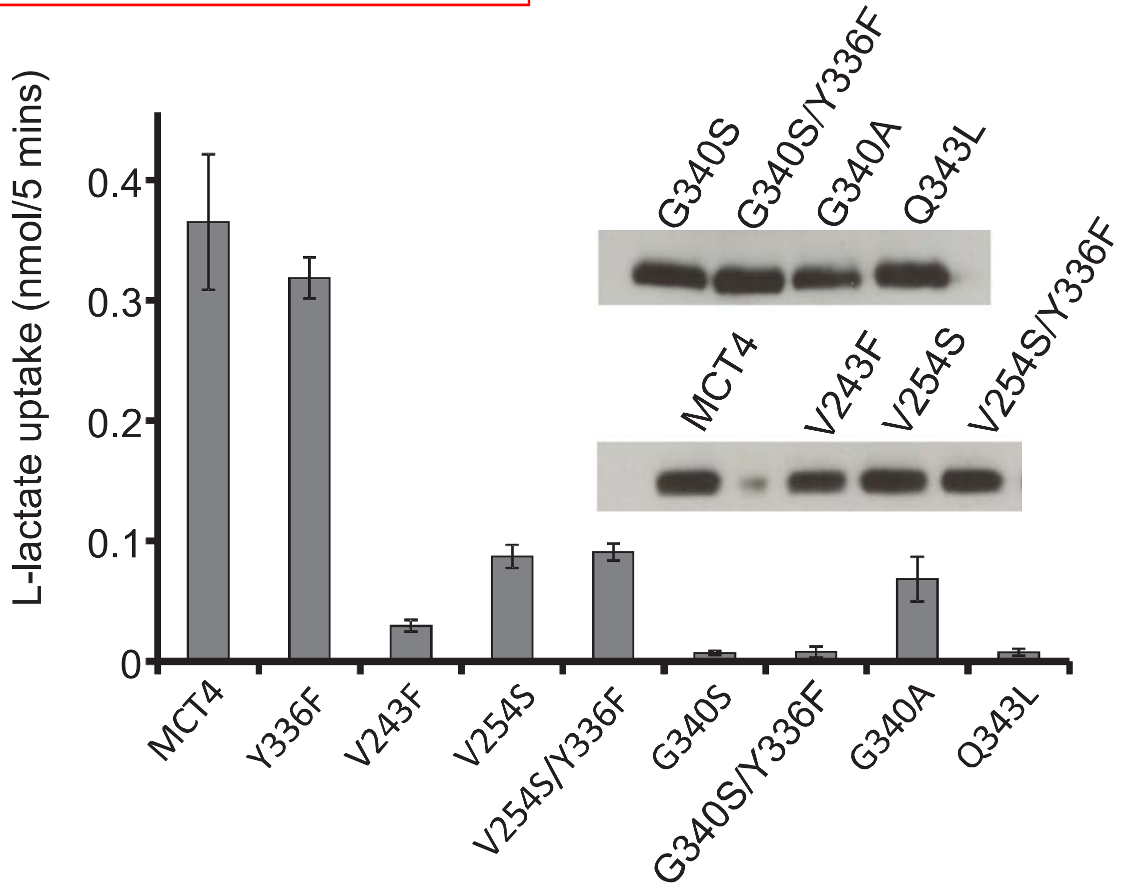
G340S
G340S/Y336F
G340A
Q343L
MCT4
V243F
V254S
V254S/Y336F
L-lactate uptake (nmol/5 mins)
0.4
0.3
0.2
0.1
0
MCT4
Y336F
V243F
V254S
V254S/Y336F
G340S
G340S/Y336F
G340A
Q343L

B

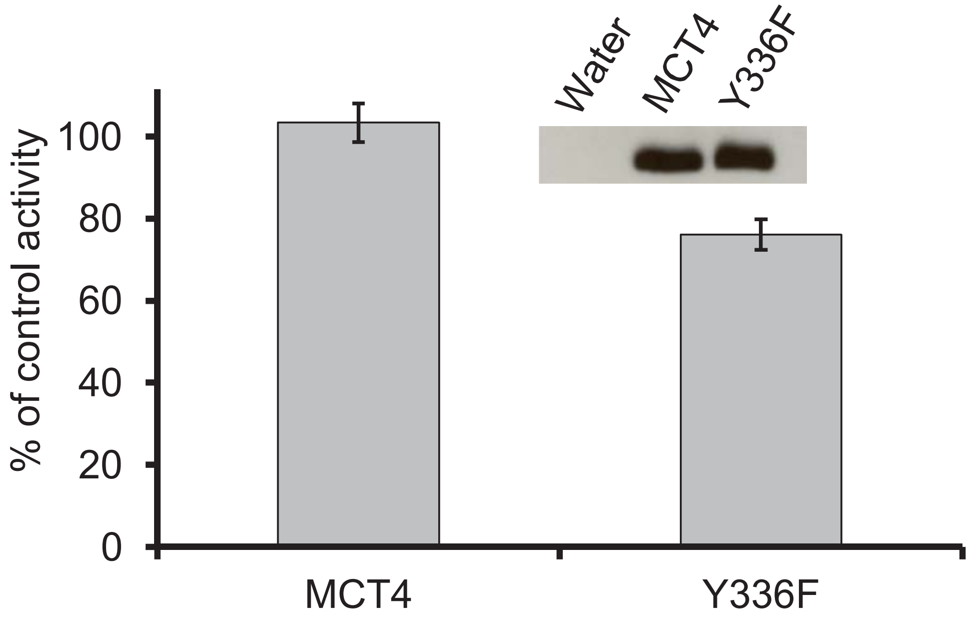
Water
MCT4
Y336F
% of control activity
100
80
60
40
20
0
MCT4
Y336F
